# Pan-coronavirus fusion inhibitors possess potent inhibitory activity against HIV-1, HIV-2, and simian immunodeficiency virus

**DOI:** 10.1080/22221751.2021.1917309

**Published:** 2021-04-29

**Authors:** Danwei Yu, Yuanmei Zhu, Hongxia Yan, Tong Wu, Huihui Chong, Yuxian He

**Affiliations:** NHC Key Laboratory of Systems Biology of Pathogens, Institute of Pathogen Biology and Center for AIDS Research, Chinese Academy of Medical Sciences and Peking Union Medical College, Beijing, People’s Republic of China

**Keywords:** Coronavirus, SARS-CoV-2, HIV-1, fusion inhibitor, lipopeptide

## Abstract

EK1 peptide is a membrane fusion inhibitor with broad-spectrum activity against human coronaviruses (CoVs). In the outbreak of COVID-19, we generated a lipopeptide EK1V1 by modifying EK1 with cholesterol, which exhibited significantly improved antiviral activity. In this study, we surprisingly found that EK1V1 also displayed potent cross-inhibitory activities against divergent HIV-1, HIV-2, and simian immunodeficiency virus (SIV) isolates. Consistently, the recently reported EK1 derivative EK1C4 and SARS-CoV-2 derived fusion inhibitor lipopeptides (IPB02 ∼ IPB09) also inhibited HIV-1 Env-mediated cell–cell fusion and infection efficiently. In the inhibition of a panel of HIV-1 mutants resistant to HIV-1 fusion inhibitors, EK1V1 and IPB02-based inhibitors exhibited significantly decreased or increased activities, suggesting the heptad repeat-1 region (HR1) of HIV-1 gp41 being their target. Furthermore, the sequence alignment and molecular docking analyses verified the target site and revealed the mechanism underlying the resistance. Combined, we conclude that this serendipitous discovery provides a proof-of-concept for a common mechanism of viral fusion and critical information for the development of broad-spectrum antivirals.

## Introduction

Infection of many enveloped viruses, including HIV-1 and SARS-CoV-2, requires membrane fusion with target cells, which is mediated by viral envelope (Env) glycoproteins composed of a receptor-binding surface subunit and a transmembrane fusion protein[1,2]. In a general mechanism, the fusion protein folds its N- and C-terminal heptad repeat (HR1 and HR2, respectively) regions into a six-helical bundle (6-HB) structure to juxtapose the viral and cell membranes for fusion [3]. Peptides derived from the HR1 and HR2 sequences can block 6-HB formation thus possessing antiviral activity. In the case of HIV-1, its fusion protein is gp41 and the HR2 peptide T20 remains the only membrane fusion inhibitor clinically available for the treatment of viral infection, which is used in combination HIV-1 therapy; however, T20 has relatively low anti-HIV activity and a genetic barrier to inducing drug resistance, calling for novel HIV-1 fusion inhibitors with improved pharmaceutical profiles [4,5]. In the past decade, we have devoted to study the mechanism of gp41-dependent HIV-1 fusion and achieved significant findings, and meanwhile, we have also developed a group of lipopeptides with ultrapotent anti-HIV activity, with one (Lipovertide) being advanced to clinical trials (NCT04592315) [6–11]. While a native HR2 peptide can block viral entry from cell surface but not entry through endosomal pathway, a lipopeptide also enables activity against viruses that are taken up via endocytosis, thus being a more suitable strategy for developing fusion inhibitors against coronaviruses (CoVs). In the outbreak of COVID-19 [12,13], we took immediate action to develop therapeutic fusion inhibitors against SARS-CoV-2, reporting a lipopetide IPB02 designed with the HR2 sequence of the S2 fusion protein [14]. Significantly, IPB02 and its derivatives exhibited robust cross-inhibitory activities against divergent human CoVs, including SARS-CoV, MERS-CoV, HCoV-229E, and HCoV-NL63 [15].

Before the emergence of SARS-CoV-2, EK1 peptide, which was originally designed with the HR2 sequence of HCoV-OC43, was also reported with a broad-spectrum anti-CoV activity [16]. As anticipated, EK1 and its lipid derivatives displayed inhibitory activities against SARS-CoV-2 [17]. By conjugating cholesterol to the C-terminal of EK1, we generated a lipopeptide termed EK1V1, which inhibited divergent CoVs more efficiently [15]. In a recent study for HIV-1, we surprisingly found that the control peptides EK1 and EK1V1 also inhibited HIV-1 infection. Considering that HIV-1 and emerging human CoVs share structural and functional similarities in fusion proteins (gp41 and S2), our finding revealed novel insights into a common mechanism of viral fusion and open an avenue for developing broad-spectrum antivirals. Thus, we next focused on characterizing the newly designed pan-CoV fusion inhibitors for their anti-HIV functionality and the mechanism underlying the inhibitory activity and resistance. We demonstrated that EK1- and IPB02-based lipopeptides were highly potent inhibitors of divergent HIV-1, HIV-2, and simian immunodeficiency virus (SIV). By applying a panel of HIV-1 mutants that are resistant to T20 and several new-generations of HIV-1 fusion inhibitors, our results suggested the HR1 of gp41 being a target site by the CoV fusion inhibitors. Further sequence alignment and molecular docking analyses verified the target and revealed the interactions in detail. We describe and discuss our findings as follows.

## Materials and methods

### Plasmids and cells

Plasmids encoding the “global panel” HIV-1 Envs (subtypes A, B, C, G, A/C, A/E, and B/C), molecular clones of HIV-2 (ROD and ST), and TZM-bl cells were obtained through the AIDS Reagent Program, Division of AIDS, NIAID, NIH. Plasmids encoding DSP_1–7_ and 293FT cells stably expressing CXCR4/CCR5/DSP_8–11_ were a kind gift from Zene Matsuda at the Institute of Medical Science of the University of Tokyo (Tokyo, Japan). Two plasmids encoding simian immunodeficiency virus (SIV) Env (mac239 and smmPBj) were kindly gifted by Jianqing Xu at the Shanghai Public Health Clinical Center, Fudan University, China. Plasmids encoding the HA protein of two avian influenza viruses (H5N1 and H7N9) were kindly gifted by Paul Zhou at the Institute Pasteur of Shanghai, Chinese Academy of Sciences, China. Plasmids encoding the G and F proteins of respiratory syncytial virus (RSV) were kindly provided by Jin-sheng He at the Beijing Jiaotong University, China. HEK293T, MDCK, and HEp-2 cells were purchased from the American Type Culture Collection (ATCC) (Rockville, MD). Cells were cultured in a complete growth medium that consisted of Dulbecco's minimal essential medium (DMEM) supplemented with 10% foetal bovine serum, 100 U/ml of penicillin–streptomycin, 2 mM l-glutamine, 1 mM sodium pyruvate, and 1× MEM nonessential amino acids (Gibco/Invitrogen, USA) and were maintained at 37°C in 5% CO_2_.

### Peptide and lipopeptide fusion inhibitors

The peptides and lipopeptides were described in the previous studies [14,15]. Briefly, EK1, IPB01, and T20 were synthesized on rink amide 4-methylbenzhydrylamine (MBHA) resin using a standard solid-phase 9-flurorenylmethoxycarbonyl (FMOC) protocol. EK1V1 and IPB02 and its truncated versions (IPB03 ∼IPB09) were synthesized by conjugating cholesterol succinate monoester to the side chain of a C-terminal lysine residue, and the lipopeptide EK1C4 was synthesized by adding bromoacetic acid cholesterol to the side chain of a C-terminal cysteine via chemoselective thioether conjugation. All peptides were acetylated at the N-terminus prior to resin cleavage, followed by purification by reverse-phase high-performance liquid chromatography (HPLC) and characterized with mass spectrometry.

### Inhibition of HIV-1 Env-mediated cell–cell fusion

Inhibitory activity of peptides or lipopeptides on HIV-1 Env-driven cell–cell fusion was determined by a dual split-protein (DSP)-based cell–cell fusion assay as described previously [6]. Briefly, a total of 1.5 × 10^4^ HEK293 T cells (effector cells) were seeded on a 96-well plate and incubated at 37°C overnight, and then they were cotransfected with an HIV-1 Env (gp160)-expressing plasmid and a DSP_1-7_ plasmid. On the next day, effector cells were added with a serially 3-fold diluted peptide or lipopeptide and incubated for 1 h; 3 × 10^4^ 293FT cells stably expressing CXCR4/CCR5 and DSP_8-11_ (target cells) were resuspended in prewarmed culture medium that contains EnduRen live-cell substrate (Promega) at a final concentration of 17 ng/mL and then transferred to the effector cell wells at equal volumes. The mixed cells were spun down to maximize cell–cell contact and incubated for 2 h. Luciferase activity was determined using a luminescence counter (Promega, Madison, WI, USA) and 50% inhibitory concentration (IC_50_) of an inhibitor was calculated using GraphPad Prism software (GraphPad Software Inc., San Diego, CA, USA).

### Inhibition of pseudovirus infection

Inhibitory activity of fusion inhibitors on diverse pseudoviruses was determined by a single-cycle infection assay as described previously [6]. In brief, pseudovirus for HIV-1, SIV, H5N1, H7N9, RSV, or VSV was generated by cotransfecting HEK293 T cells with an Env-encoding plasmid and a backbone plasmid (pNL4-3.luc.RE) that encodes an Env-defective, luciferase reporter-expressing HIV-1 genome. After transfection 48 h, virus-containing supernatants were harvested and 50% tissue culture infectious dose (TCID_50_) was measured. Peptides or lipopeptides were prepared in 3-fold dilutions, mixed with 100 TCID_50_ of viruses. After incubation for 1 h at room temperature, one hundred microliters of the mixture were added to target cells (TZM-bl for HIV-1, SIV and VSV; MDCK for H5N1 and H7N9; HEp-2 for RSV) that were plated at 10^4^ cells/well and then incubated for 48 h at 37°C. The cells were harvested and lysed in reporter lysis buffer, and luciferase activity was measured using luciferase assay reagents and a luminescence counter (Promega), and IC_50_ values were calculated as described above.

### Inhibition of replication-competent HIV-2 isolates

Inhibitory activity of fusion inhibitors on two replication-competent HIV-2 (ROD and ST) isolates was determined as described previously [6]. Briefly, viral stocks were prepared by transfecting viral molecular clones into HEK293T cells. Culture supernatants were harvested 48 h posttransfection, and TCID_50_ was measured in TZM-bl cells. An inhibitor was prepared in 3-fold dilutions, mixed with 100 TCID_50_ of viruses, and then incubated 1 h at room temperature. The mixture was added to TZM-bl cells (10^4^/well in a 100 μl volume) and incubated for 48 h at 37°C. The cells were harvested and lysed in reporter lysis buffer, and luciferase activity was measured using luciferase assay reagents and a luminescence counter, and IC_50_ values were accordingly calculated.

### Cytotoxicity of coronavirus fusion inhibitors

The cytotoxicity of fusion inhibitors on HEK293T cells and TZM-bl cells was measured using a CellTiter 96 AQueous One Solution cell proliferation assay (Promega). In brief, 50-μl volumes of lipopeptides at graded concentrations were added to cells, which were seeded on a 96-well tissue culture plate (1 × 10^4^ cells per well). After incubation at 37°C for 2 days, 20 μl of CellTiter 96 AQueous One solution reagent was added into each well and incubated 2 h at 37°C. The absorbance was measured at 490 nm using a SpectraMax M5 microplate reader (Molecular Devices, San Jose, CA, USA), and cell viability (percentage) was calculated.

### Size-exclusion chromatography

The binding between EK1 peptide and a target mimic peptide derived from the HR1 sequence of gp41 was analysed by size-exclusion chromatography. In brief, EK1 was mixed with N42, N36 or N38 (molar ratio of 1:1) in 0.05M sodium phosphate (pH 7.2) at a final concentration of 0.2 mM, and then incubated at 37°C for 30 min. The mixture was applied to the Superdex-75 10/300 GL (GE Healthcare, Piscataway, NJ, USA) equilibrated with 50 mM sodium phosphate and eluted at 0.8 ml/min, and fractions were monitored at 214 nm.

### Molecular simulation and peptide docking

The trimeric gp41 HR1 helices containing more intact sequences were built using molecular simulation from two crystal gp41 core structures (PDB: 2X7R and PDB: 3VGX), while EK1 was derived from its crystal structure bound to a target mimic peptide determined recently (PDB: 5ZUV). Docking of the peptide ligands to HIV-1 HR1 was performed using the Discovery studio package (DS). The Prepare Protein tool in DS is used to pretreat the receptor protein and ligand protein, including the determination of protonation state and hydrogenation. Dock Proteins (ZDOCK) tool in DS was used to perform docking procedures for ligand and receptor proteins. According to the calculation results, the best 100 rated conformations in the 10 largest clusters were selected for optimization. After optimization, according to E_RDock rating, the binding conformation with the lowest energy score was selected as the final ligand binding conformation. Finally, open the interaction analysis tool Analyse Protein Interface for analysis.

## Results

### Identification of EK1-based lipopeptides as potent HIV-1 inhibitors

**Figure 1.**
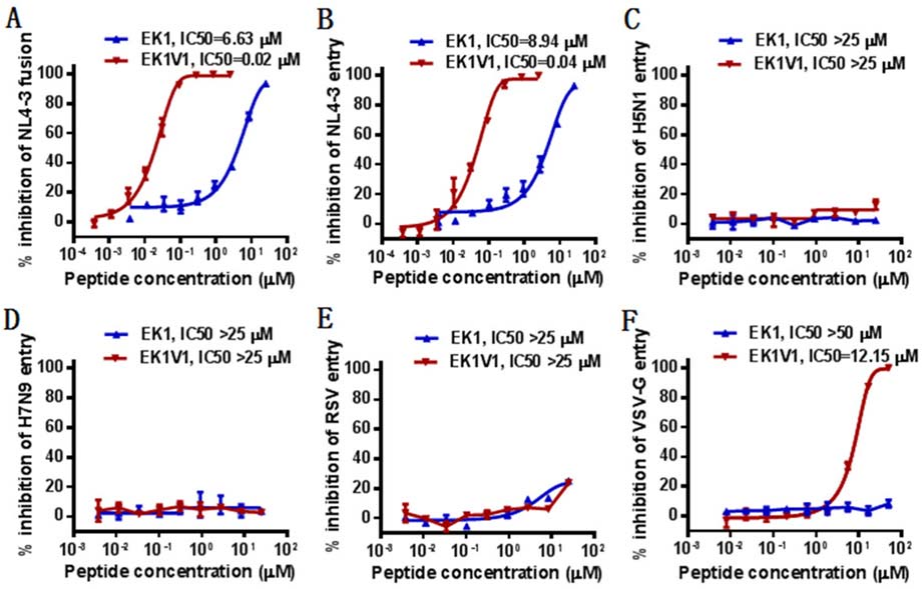
Cross-inhibitory activity of coronavirus fusion inhibitors against HIV-1. (A) Inhibition of EK1 and EK1V1 on HIV-1_NL4-3_ Env-mediated cell–cell fusion was determined by a dual-split protein (DSP)-based cell fusion assay. The inhibitory activities of EK1 and EK1V1 against the pseudoviruses of HIV-1_NL4-3_ (B), H5N1 (C), H7N9 (D), RSV (E), and VSV (F), were determined by a single-cycle infection assay. Both the cell fusion and pseudovirus infection assays were repeated at least three times, and data are expressed as means ± standard deviations (SD) while the mean IC_50_ values are shown.

In an experiment for HIV-1 project, we used the peptide EK1 and its lipid derivative EK1V1 as control inhibitors. Very surprisingly, we found that both EK1 and EK1V1 efficiently inhibited HIV-1 infection. As determined by a dual-split protein (DSP)-based cell–cell fusion assay, EK1 and EK1V1 inhibited HIV-1_NL4-3_ Env-mediated cell–cell fusion with mean IC_50_ values of 6.63 and 0.02 μM, respectively (Figure 1(A)). As determined by a single-cycle infection assay, EK1 and EK1V1 inhibited HIV-1_NL4-3_ pseudovirus infection with mean IC_50_ of 8.94 and 0.04 μM, respectively (Figure 1(B)). When the testing was expanded, both EK1 and EK1V1 inhibitors had no appreciable activity in inhibiting the pseudoviruses of two avian influenza viruses (H5N1 and H7N9) and respiratory syncytial virus (RSV); however, EK1V1, but not EK1, dose-dependently blocked the vesicular stomatitis virus pseudoytpe (VSV-G) with an IC_50_ of 12.15 μM (Figure 1(C–F)). A panel of primary HIV-1 Envs, which represent the worldwide AIDS epidemic [18], was further applied to characterize the inhibitory activity of EK1 and EK1V1. As shown in Table 1, while EK1 failed to inhibit the Env-based cell fusion and pseudovirus infection, EK1V1 possessed highly potent inhibitory activities. Specifically, EK1V1 inhibited the cell fusion with a mean IC_50_ at 0.17 μM and inhibited divergent HIV-1 pseudoviruses with a mean IC_50_ at 0.9 μM.

**Table 1.** Cross-inhibitory activity of EK1 and EK1V1 against different subtypes of HIV-1 isolates (IC_50_ ± SD, μM)^a^.

HIV-1 Env	Subtype	Cell fusion (IC50±SD, μM)		Pseudovirus infection (IC50±SD, μM)	
		EK1	EK1V1	EK1	EK1V1
398-F1_F6_20	A	> 5	0.17 ± 0.01	> 12.5	1.2 ± 0.23
TRO.11	B	> 5	0.68 ± 0.17	> 12.5	1.35 ± 0.28
X2278_C2_B6	B	> 5	0.05 ± 0.004	> 12.5	0.88 ± 0.12
JRFL	B	> 5	0.05 ± 0.001	> 12.5	0.11 ± 0.02
SF162	B	> 5	0.05 ± 0.01	> 12.5	0.1 ± 0.011
CE703010217_B6	C	> 5	0.04 ± 0.01	> 12.5	0.2 ± 0.03
HIV_25710-2.43	C	> 5	0.17 ± 0.03	> 12.5	0.71 ± 0.08
CE1176_A3	C	> 5	0.35 ± 0.1	> 12.5	0.89 ± 0.02
X1632-S2-B10	G	> 5	0.04 ± 0.01	> 12.5	0.85 ± 0.17
246_F3_C10_2	A/C	> 5	0.05 ± 0.01	> 12.5	1.03 ± 0.19
CNE8	A/E	> 5	0.49 ± 0.09	> 12.5	1.23 ± 0.12
CNE55	A/E	> 5	0.13 ± 0.03	> 12.5	3.02 ± 0.48
CH119.10	B/C	> 5	0.02 ± 0.004	> 12.5	0.57 ± 0.04
BJOX002000.03.2	B/C	> 5	0.07 ± 0.01	> 12.5	0.5 ± 0.03
Mean		> 5	0.17	> 12.5	0.9

^a^ The assay was performed in triplicate and repeated three times, and data are expressed as means ± SD.

### EK1V1 is a potent inhibitor of divergent HIV-2 and SIV isolates

**Figure 2.**
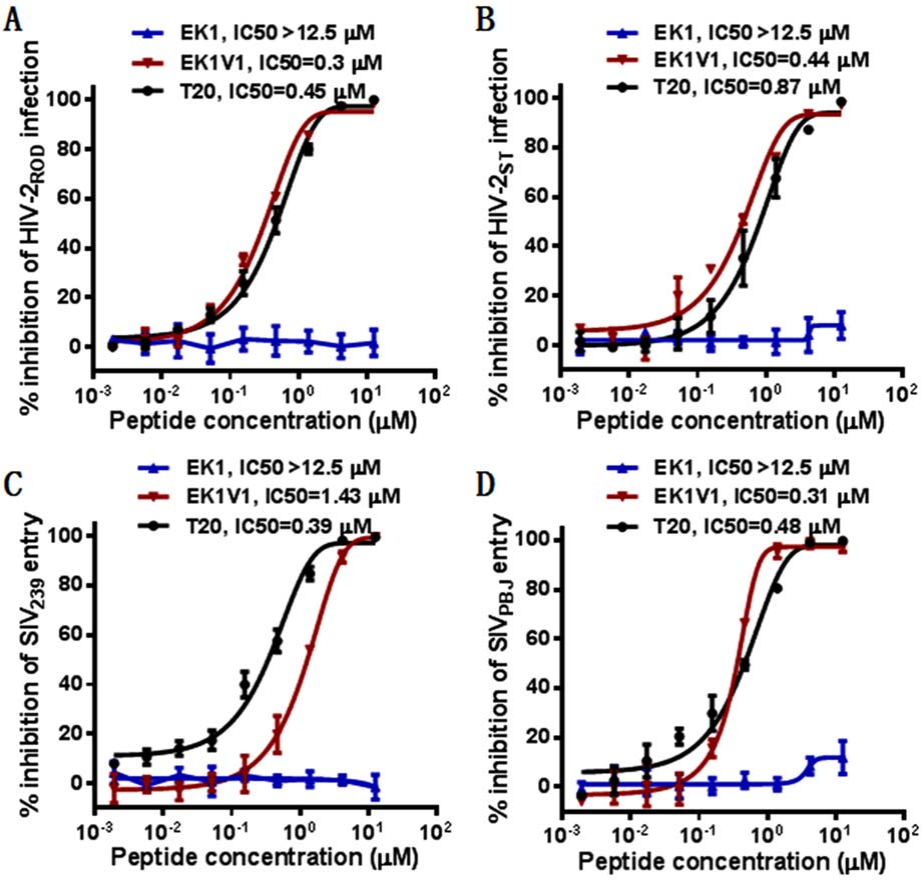
Cross-inhibitory activity of EK1 and EK1V1 on HIV-2 and simian immunodeficiency virus (SIV). Inhibition of replication-competent HIV-2_ROD_ (A) and HIV-2_ST_ (B) was determined by P24-based assay and inhibition of SIV_293_ (C) and SIV_PBJ_ (D) pseudoviruses was determined by pseudovivirus-based single-cycle infection assay. The experiments were repeated three times, and data are expressed as means ± SD while the mean IC_50_ values are shown.

We were interested to know whether EK1 and EK1V1 were active against HIV-2 and SIV isolates, which genetically and functionally share similarities with HIV-1 isolates. To this end, two replication-competent HIV-2 (ROD and ST) and two SIV pseudoviruses (239 and PBJ) were applied to measure the inhibitory activity of EK1 and EK1V1 along with the peptide drug T20. As shown in Figure 2, EK1 had no inhibitory activity on HIV-2 and SIV infections at a high concentration (12.5 μM); however, EK1V1 inhibited HIV-2_ROD_ and HIV-2_ST_ with IC_50_ of 0.3 and 0.44 μM, respectively and inhibited SIV_239_ and SIV_PBJ_ with IC_50_ of 1.43 and 0.31 μM, respectively. In comparison, T20 inhibited the four viruses with IC_50_ of 0.45, 0.87, 0.39, and 0.48 μM, respectively. Taken together, these results demonstrated that EK1V1 is a potent inhibitor of divergent HIV-1, HIV-2, and SIV isolates, validating a proof-concept for the development of broad-spectrum viral fusion inhibitors.

### Characterization of EK1C4 and SARS-CoV-2 derived fusion inhibitor lipopeptides

**Figure 3.**
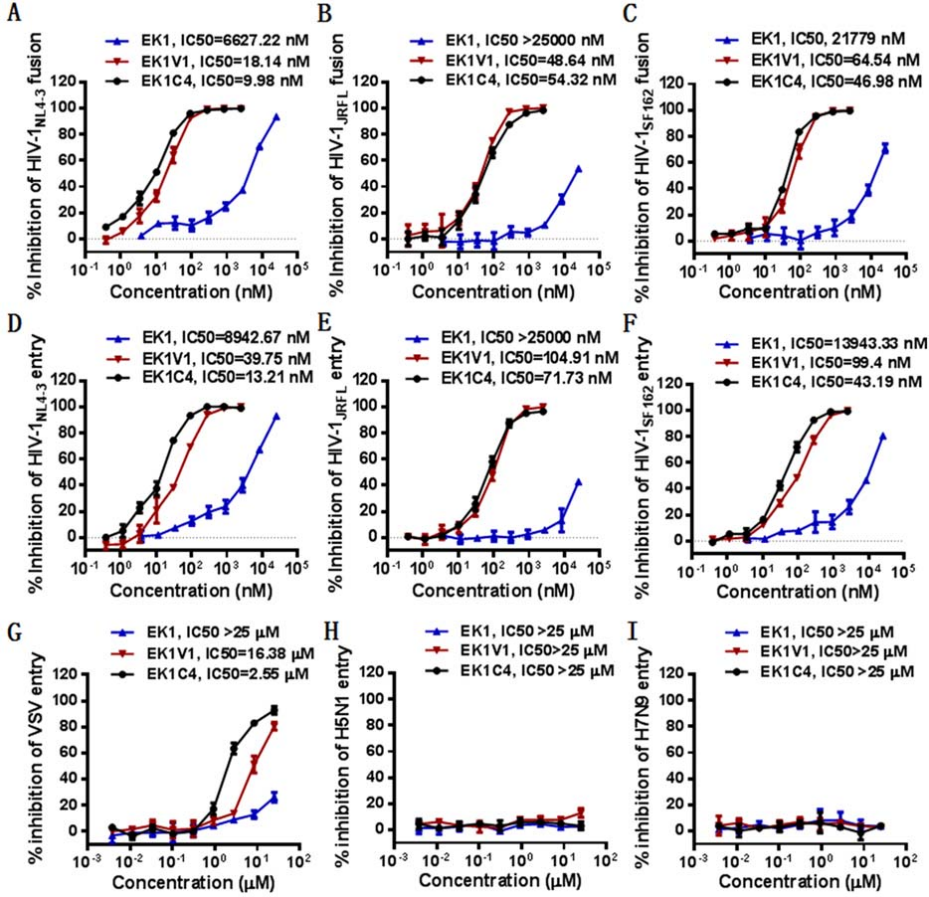
Cross-inhibitory activity of EK1C4 on HIV-1 cell fusion and pseudovirus infection. Inhibition of the Env-mediated cell–cell fusion activity of HIV-1_NL4-3_ (A), HIV-1_JRFL_ (B), and HIV-1_SF162_ (C) was determined by a DSP-based cell–cell fusion assay. Inhibition of HIV-1_NL4-3_ (D), HIV-1_JRFL_ (E), and HIV-1_SF162_ (F), VSV-G (G), H5N1 (H), and H7N9 (I) pseudoviruses were determined by a single-cycle infection assay. The experiments were repeated three times, and data are expressed as means ± SD while the mean IC_50_ values are shown.

Following our studies, a new EK1-based lipopeptide, EK1C4, was reported with significantly improved anti-CoV activity [17]. Comparing to EK1V1, EK1C4 was introduced with two linkers (GSGSG and PEG4) between the peptide sequence and cholesterol molecule. Herein, we determined its anti-HIV activity in comparison with EK1 and EK1V1. As shown in Figure 3, EK1C4 displayed comparable or slightly increased activity over EK1V1 in inhibiting three HIV-1 isolates (NL4-3, JRFL, and SF162) in terms of Env-based cell fusion and pseudoviruses, verifying the cross-reactive inhibition. Next, we characterized the anti-HIV activity of a panel of SARS-CoV-2 derived fusion inhibitor lipopeptides [14]. Consistently, IPB02 and its truncated versions also possessed high potencies on HIV-1_NL4-3_ cell fusion and infection (Table 2). Interestingly, while IPB02 inhibited the cell fusion and pseudovirus with IC_50_ of 0.05 and 0.11 μM, respectively, the N-terminally truncated IPB04 and C-terminally truncated IPB08 maintained similar antiviral activities, informing the structure–activity relationship (SAR) of the anti-CoV lipopeptides on HIV-1. In order to verify the specificity, the cytotoxicity of six representative lipopeptides including EK1, EK1V1, EK1C4, IPB02, IPB04, and IOB08 was determined, and none of them at a concentration of 10 or 25 μM showed significant cytotoxic effects on HEK293T and TZM-bl cells (Fig. S1).

**Table 2.** Cross-inhibitory activity of EK1- and IPB02-based CoV fusion inhibitors against HIV-1_NL4-3_ Env-mediated cell–cell fusion and pseudovirus infection^a^.

CoV inhibitor	Sequence structure	IC50 ± SD (μM)	
		Env cell fusion	HIV pseudovirus
EK1	SLDQINVTFLDLEYEMKKLEEAIKKLEESYIDLKEL	>5	>5
EK1V1	SLDQINVTFLDLEYEMKKLEEAIKKLEESYIDLKELK(Chol)	0.02 ± 0.005	0.04 ± 0.004
EK1C4	SLDQINVTFLDLEYEMKKLEEAIKKLEESYIDLKEL-GSGSG-PEG4-C(Chol)	0.01 ± 0.001	0.01 ± 0.002
IPB01	ISGINASVVNIQKEIDRLNEVAKNLNESLIDLQEL	>25	>25
IPB02	ISGINASVVNIQKEIDRLNEVAKNLNESLIDLQELK(Chol)	0.05 ± 0.02	0.11 ± 0.01
IPB03	INASVVNIQKEIDRLNEVAKNLNESLIDLQELGK(Chol)	0.04 ± 0.01	0.2 ± 0.07
IPB04	SVVNIQKEIDRLNEVAKNLNESLIDLQELGK(Chol)	0.09 ± 0.03	0.18 ± 0.05
IPB05	IQKEIDRLNEVAKNLNESLIDLQELGK(Chol)	0.2 ± 0.06	0.62 ± 0.11
IPB06	IDRLNEVAKNLNESLIDLQELGK(Chol)	4.6 ± 1.26	0.37 ± 0.01
IPB07	IQKEIDRLNEVAKNLNESLIDLQELGKYEQYIK(Chol)	0.73 ± 0.2	0.25 ± 0.03
IPB08	ISGINASVVNIQKEIDRLNEVAKNLNESLIK(Chol)	0.04 ± 0.02	0.16 ±0.04
IPB09	SVVNIQKEIDRLNEVAKNLNESLIK(Chol)	0.09 ± 0.04	1.25 ± 0.37

^a^ The assay was performed in triplicate and repeated three times, and data are expressed as means ± SD.

### Inhibitory activity of Pan-CoV fusion inhibitors on HIV-1 mutants resistant to fusion inhibitors

The resistance mutations for T20 and newly designed HIV-1 fusion inhibitors are predominantly mapped to the inhibitor-binding sites on the HR1 of gp41 [19–21]. In order to exploit the mechanism underlying the cross-inhibition, here we sought to define whether EK1V1 and IPB02-based inhibitors were effective against various HIV-1 variants bearing the resistant mutations in the HR1 site. A panel of mutant HIV-1_NL4-3_ pseudoviruses was prepared and used in the single-cycle infection assay. As shown in Table 3, while some HIV-1 mutants had similar sensitivities to diverse CoV fusion inhibitors, multiple mutations conferred various degrees of resistance. Interestingly, the L568R mutation in the deep HR1 pocket site, which was selected by short-peptide HIV fusion inhibitors mainly targeting the pocket, rendered the virus more sensitive to EK1V1 and the IPB02 derivatives, while the HIV-1 variant with E560 K in the middle HR1 region displayed increased sensitivity to EK1V1 only. The results validated the gp41 HR1 being a target site by EK1- and IPB02-based inhibitors.

**Table 3.** Cross-inhibitory activity of EK1V1 and IPB02-based CoV fusion inhibitors against HIV-1_NL4-3_ mutants resistant to HIV-1 fusion inhibitors^a^.

HIV-1_NL4-3_	EK1V1		IPB02		IPB04		IPB06		IPB08		IPB09	
	IC_50_	n-fold	IC_50_	n-fold	IC_50_	n-fold	IC_50_	n-fold	IC_50_	n-fold	IC_50_	n-fold
WT	0.04 ± 0.002	1	0.13 ± 0.05	1	0.25 ± 0.04	1	1.03 ± 0.14	1	0.11 ± 0.04	1	0.3 ± 0.07	1
D547G	0.04 ± 0.002	1	0.16 ± 0.06	1.23	0.32 ± 0.05	1.28	1.7 ± 0.62	1.65	0.15 ± 0.06	1.36	0.38 ± 0.08	1.27
I548T	0.24 ± 0.01	**6**	1.64 ± 0.53	**12.62**	1.16 ± 0.43	**4.64**	>5	**>4.85**	0.47 ± 0.09	**4.27**	1.56 ± 0.53	**5.2**
V549A	0.11 ± 0.02	2.75	0.41 ± 0.12	**3.15**	0.39 ± 0.08	1.56	>5	**>4.85**	0.19 ± 0.07	1.73	0.85 ± 0.39	2.83
V549M	0.28 ± 0.05	**7**	0.24 ± 0.09	1.85	0.22 ± 0.05	0.88	2.7 ± 0.58	2.62	0.19 ± 0.04	1.73	0.67 ± 0.21	2.23
Q551H	0.02 ± 0.01	0.5	0.08 ± 0.03	0.62	0.1 ± 0.03	0.4	2.57 ± 0.49	2.5	0.09 ± 0.04	0.82	0.27 ± 0.13	0.9
N554K	0.18 ± 0.01	**4.5**	2.29 ± 0.42	**17.62**	0.72 ± 0.28	2.88	>5	**>4.85**	3.31 ± 0.61	**30.09**	>5	**>16.67**
E560K	0.01 ± 0.003	**0.25**	2.6 ± 0.24	**20**	0.93 ± 0.29	**3.72**	2.24 ± 0.62	2.17	0.46 ± 0.19	**4.18**	>5	**>16.67**
L568R	0.004 ± 0.0004	**0.1**	0.01 ± 0.002	**0.08**	0.04 ± 0.02	**0.16**	0.32 ± 0.08	**0.31**	0.01 ± 0.004	**0.09**	0.02 ± 0.002	**0.07**
D547S/V549M	0.12 ± 0.01	**3**	0.44 ± 0.07	**3.38**	0.53 ± 0.17	2.12	>5	**>4.85**	0.32 ± 0.13	2.91	0.98 ± 0.34	**3.27**
I548T/N554K	0.36 ± 0.04	**9**	>5	**38.46**	>5	**20**	>5	**>4.85**	>5	**>45.45**	>5	**>16.67**
V549A/N553T	0.31 ± 0.03	**7.75**	0.49 ± 0.16	**3.77**	0.56 ± 0.22	2.24	>5	**>4.85**	0.21 ± 0.08	1.91	0.8 ± 0.34	2.67

^a^ The assay was performed in triplicate and repeated three times, and data are expressed as means ± SD. The fold changes (n-fold) greater than 3 are marked in bold.

### Sequence alignment and molecular docking analyses

**Figure 4.**
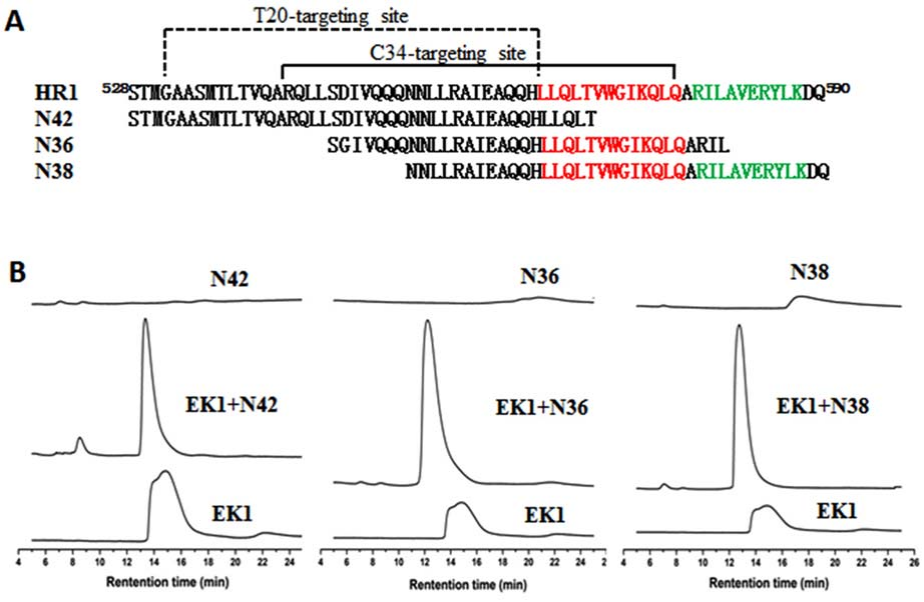
Interaction of EK1 peptide with the gp41 HR1-derived peptides. (A) The HR1 sequence of HIV-1 gp41 and HR1-derived target mimic peptides. The pocket-1 and pocket-2 forming sequences on HR1 are marked in red and green, respectively. (B) Interaction of EK1 peptide with the gp41 HR1-derived peptides was analysed by size-exclusion chromatography.

To elucidate the binding sites of the CoV fusion inhibitors on HIV-1 gp41 in detail, we initially worked to solve the crystal structure of EK1 or EK1V1 complexed with target mimic peptides. As indicated by size-exclusion chromatography (Figure 4), EK1 could form peptide complexes with each of three gp41 HR1-derived overlapping peptides (N42, N36, and N38), confirming its interaction with the gp41 HR1 site. The peptide complexes were collected for crystallization trials, but so far we have not obtained the crystals suitable for collecting the structural data. Thus, we sought to exploit the EK1-binding sites on gp41 by bioinformatics methods. As shown in Fig. S2A, the extracellular domains of the SARS-CoV-2 S2 and HIV-1 gp41 fusion proteins share structural and functional similarities, including N-terminal fusion peptide (FP), HR1, loop region, HR2, WYF motif, and transmembrane (TM) domain, and folding a hairpin structure between HR1 and HR2 is required for membrane juxtaposition and fusion. When the HR1 sequences of the HIV-1_NL4-3_ gp41 and S2 were compared by alignment, the S2 HR1 pocket-forming sequence was finely aligned with the gp41 pocket 1-forming sequence and the predicted EK1-binding sequences on gp41 overlap the T20-resistant site, pocket-1, and pocket-2 (Fig. S2B). Interestingly, the HIV-2_ROD_ and SIV_239_ HR1 sequences even possess more conservative residues with the S2 HR1 relative to the HIV-1_NL4-3_ HR1 core site (Fig. S2C).

**Figure 5.**
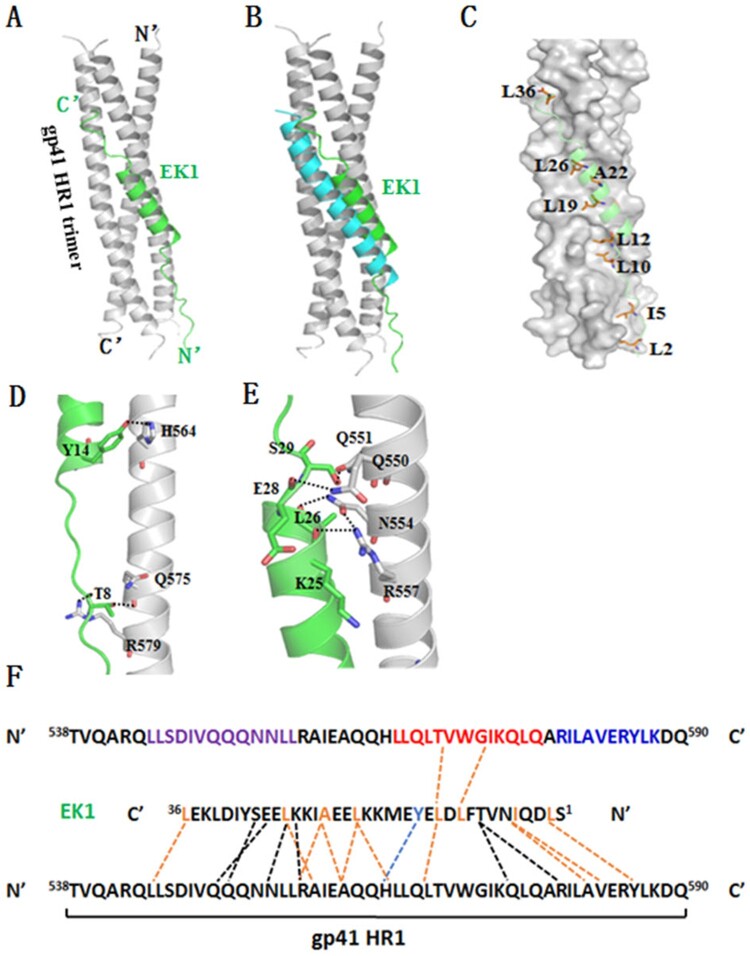
Binding model of EK1 peptide with the HR1 of HIV-1 gp41 by molecular docking. The residues involving hydrogen bonds and hydrophobic interaction are shown as stick models with labels. Hydrogen bonds and PI-bond are indicated in dashed lines. (A) A ribbon model of EK1/HR1 structure, in which the HR1 trimer is coloured in grey and EK1 is in green. (B) Superimposing of EK1 with the gp41 HR2 peptide C34 (in cyan). (C) A group of hydrophobic residues of EK1 make extensive contacts with the gp41 HR1 surface critically determining the inhibitor binding. (D) At the extended N terminal of EK1, T8 donates a hydrogen to the gp41 Gln-575 while it accepts a hydrogen from the gp41 Arg-579. At the beginning of EK1 helix portion, a PI-bond is formed between Y14 of EK1 and His-564 of gp41. (E) Around the end of the EK1 helix, a hydrogen-bond network encompasses four pairs of interactions: the long side chain of Arg-557 donates a hydrogen bond to the O_atom of K25, the side chain of Asn-554 donates a hydrogen bond to the O_atom of L26, the side chain of Gln-550 donates a hydrogen bond to the O_atom of E28, and the side chain of Gln-551 accepts a hydrogen bond from S29. (F) Sequence illustration of EK1 binding modelled by molecular docking. A single EK1 peptide interacting with two NHR helices is shown in a sequence map. The dashed black lines indicate the interhelical hydrogen bonds, the dashed blue line indicates a pi-bond, and the dashed orange lines indicate hydrophobic interactions. The sequences mediating T20 resistance, pocket-1 site, and pocket-2 site are marked in purple, red, and blue, respectively

Next, we performed molecular docking for EK1 inhibitor. The trimeric gp41 HR1 helices containing more intact sequences were built using molecular simulation from two crystal gp41 core structures (PDB accession numbers: 2X7R and 3VGX), while EK1 was derived from its crystal structure bound to a target mimic peptide determined recently (PDB accession number: 5ZUV). After peptide preparation, molecular docking, conformational optimization and grading, the lowest energy result pose 305 (E_RDock: −28.47) was obtained. As is shown in Figure 5(A), one EK1 peptide, which is composed of a middle helix portion and two terminal extended portions, binds into the groove of two gp41 HR1 helices in reverse parallel. Different from the gp41 core structure, in which a fully helical HR2-derived peptide inhibitor C34 is positioned in the middle site between the two HR1 helices, whereas EK1 is docked more closely to one HR1 chain (Figure 5(B)). In detail, a group of hydrophobic residues (L2, I5, L10, L12, L19, A22, L26, and L36) of EK1 make extensive contacts with the gp41 HR1 surface (Figure 5(C)), critically determining the inhibitor binding. At the extended N-terminal of EK1 (Figure 5(D)), T8 donates a hydrogen to the gp41 Gln-575 while it accepts a hydrogen from Arg-579. At the beginning of EK1 helix portion, a PI-bond is formed between Y14 of EK1 and His-564 of gp41. Around the end of the EK1 helix, a hydrogen-bond network encompasses four pairs of interactions: the long side chain of Arg-557 donates a hydrogen bond to the O_ atom of K25, the side chain of Asn-554 donates a hydrogen bond to the O_atom of L26, the side chain of Gln-550 donates a hydrogen bond to the O_ atom of E28, and the side chain of Gln-551 accepts a hydrogen bond from S29 (Figure 5(E)).

In the model, both Gln-550 and Arg-550 can interact with Glu-28, and the difference is that there is electrostatic interaction between Arg-550 and Glu-28 while hydrogen bonds between Gln-550 and Glu-28 (Fig. S3A). Histidine substitution of Gln-551 lost its hydrogen bond to Ser-29, weakening the binding with EK1. However, a PI-bond formed between the side chain of His-551 and Leu-555 (Fig. S3B). Glu-560 accepted a hydrogen bond from the side chain of His-564 (Fig. S3C). While Leu-568 can only form a hydrophobic interaction with Leu-12, Arg-568 formed three electrostatic interactions with Asp-11, Glu-13, and Glu-15, respectively, thus the virus with L568R is more sensitive to EK1 (Fig. S3D). Ile-548 is localized at the centre of HR1, the I548T mutation can break the hydrophobic core formed by three Ile-548 residues, destabilizing the HR1 helix. A hydrogen bond between Asn-554 and Leu-26 contributes to keep the stability of hydrogen bond network (Fig. S3E). Substitution of Val-549 might introduce a less hydrophobic residue or introduce a large amino acid, interfering with the hydrogen bond network, while substitution of Asn-553 causes the change of side chain location, causing steric obstruction to the hydrogen bond network (Fig. S3F). In short, the structural data analysed here confirmed the interaction of EK1 peptide with gp41 and revealed the mechanism underlying the resistance phenotype.

## Discussion

In this study, we serendipitously discovered the inhibitory activity of the broad-spectrum CoV fusion inhibitors EK1 and EK1V1 against HIV-1 infection in terms of viral Env-mediated cell–cell fusion and pseudovirus infection. As shown, EK1V1 exhibited highly potent activity in inhibiting divergent HIV-1, HIV-2, and SIV isolates. Moreover, the EK1-based EK1C4 and SARS-CoV-2 derived fusion inhibitor lipopeptides (IPB02 ∼ IPB09) also inhibited HIV-1 fusion and host entry efficiently. In a general mechanism of action, the fusion protein HR2-derived fusion inhibitor peptides target the counterpart HR1 site to competitively block the formation of viral 6-HB structure, thus inhibiting viral host entrance. In agreement with this mode, we found that EK1V1 and IPB02-based inhibitors inhibited HIV-1 variants bearing the HR1 mutations with significantly decreased or increased activities. In the absence of a crystal structure of EK1V1 or IPB02 complexed to the gp41-derived target mimic peptide, our sequence alignment and molecular docking did verify the HR1 region of HIV-1 gp41 being the target site by CoV fusion inhibitors. In short, we, for the first time, demonstrated the cross-inhibitory activity of CoV fusion inhibitors on HIV-1, HIV-2, and SIV by targeting the HR1 site.

Given the remarkable similarity in fusion mechanisms of HIV-1 and SARS-CoV that emerged in 2002 and the structural similarity of gp41 and S2 fusion proteins, several previous studies investigated whether HIV-1 fusion inhibitors could be used to inhibit the SARS-CoV S protein-mediated membrane fusion [22–24]. While the approved peptide drug T20 was specially focused, several fusion-inhibitory peptides under the preclinical studies, including C34, T1249, and a D-peptide, were also characterized. The biophysical studies showed a significant interaction between T20 and a SARS-CoV HR1-derived peptide, suggesting that T20 could inhibit the fusion of SARS-CoV with the target cells but its effect is not strong enough for application [22]. Very recently, an *in silico* drug repurposing study showed that the interaction between T20 and SARS-CoV-2 S protein was remarkably stable and caused the S2 protein residues to undergo the fewest fluctuations, thus proposing T20 as potent SARS-CoV-2 fusion inhibitor with the potential to enter the clinical trial phase of COVID-19 [25]. However, the previous studies were primarily based on the bioinformatics approaches and lacked experimental evidence. After the SARS-CoV-2 outbreak, we actually tested a panel of HIV-1 fusion inhibitors, including T20, the recently approved long-lasting albuvirtide (ABT) [26], T1249, T2635 [27], sifuvirtide (SFT) [28], and 2P23 that mainly targets the gp41 pocket site [29]. While they potently inhibited HIV-1, none was found to have inhibitory activity against SARS-CoV-2 at a concentration as high as 25 or 50 μM (Table S1). To fight against the emerging highly pathogenic human CoVs, a group of fusion inhibitor peptides or lipopeptides have been developed [14,17,30–34], but so far, as we known, no studies describe the cross-reactive inhibition of S2 fusion protein-derived inhibitors on HIV-1 infection. Thus, the presented data here offer a proof-of-concept for a common mechanism of viral fusion and important information for the development of antivirals with a broad-spectrum antiviral activity.

Previous studies identified a deep hydrophobic pocket (pocket-1) on the HR1 helices of gp41, which is formed by a cluster of 11 residues (Leu-565, Leu-566, Leu-568, Thr-569, Val-570, Trp-571, Gly-572, Ile-573, Lys-574, Leu-576, and Gln-577) and inserted by three hydrophobic residues from the HR2-derived peptides (Trp-628, Trp-631, and Ile-635) [3,4]. We and others also identified a subpocket (pocket-2), which locates immediately downstream of the pocket-1 and is formed by a cluster of seven residues, including Leu-587, Lys-588 and Glu-584 on one HR1 helix and Tyr-586, Val-583, Ala-582 and Arg-579 of another HR1 helix [35,36]. Both pockets play critical roles in the stability of the 6-HB core and offer ideal target sites for anti-HIV agents. Because T20 does not include the pocket-binding sequence, the HR2 peptide C34 has been widely used as a designing template for novel HIV-1 fusion inhibitors [5]. This work also revealed that EK1 binds to the fusion protein gp41 largely overlapping with the C34 binding site and extends to the pocket-2 region. Considering that none of the current HIV-1 fusion inhibitors targets the pocket-2, EK1- and IPB02-based inhibitors do provide valuable tools for exploring the structure and function relationship of gp41 and for developing novel HIV-1 fusion inhibitors that also target the pocket-2 site.

## Supplementary Material

Supplemental Material

## Data Availability

All data are fully available without restriction.
