# Supplementary figures and images for: Pan-coronavirus fusion inhibitors possess potent inhibitory activity against HIV-1, HIV-2, and simian immunodeficiency virus
Source: Emerg Microbes Infect. 2021 Apr 29;10(1):810–21. doi: 10.1080/22221751.2021.1917309 (PMC8812798; doi:10.1080/22221751.2021.1917309)

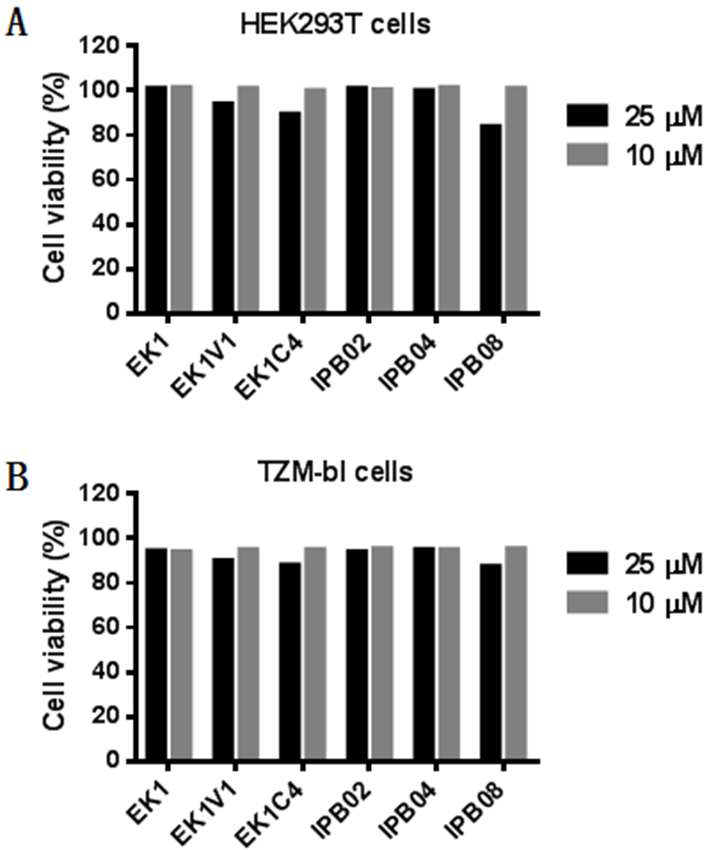

Supplement: Supplemental Material [file TEMI_A_1917309_SM8534.zip › Supplementary files/Figure-S1.tif]

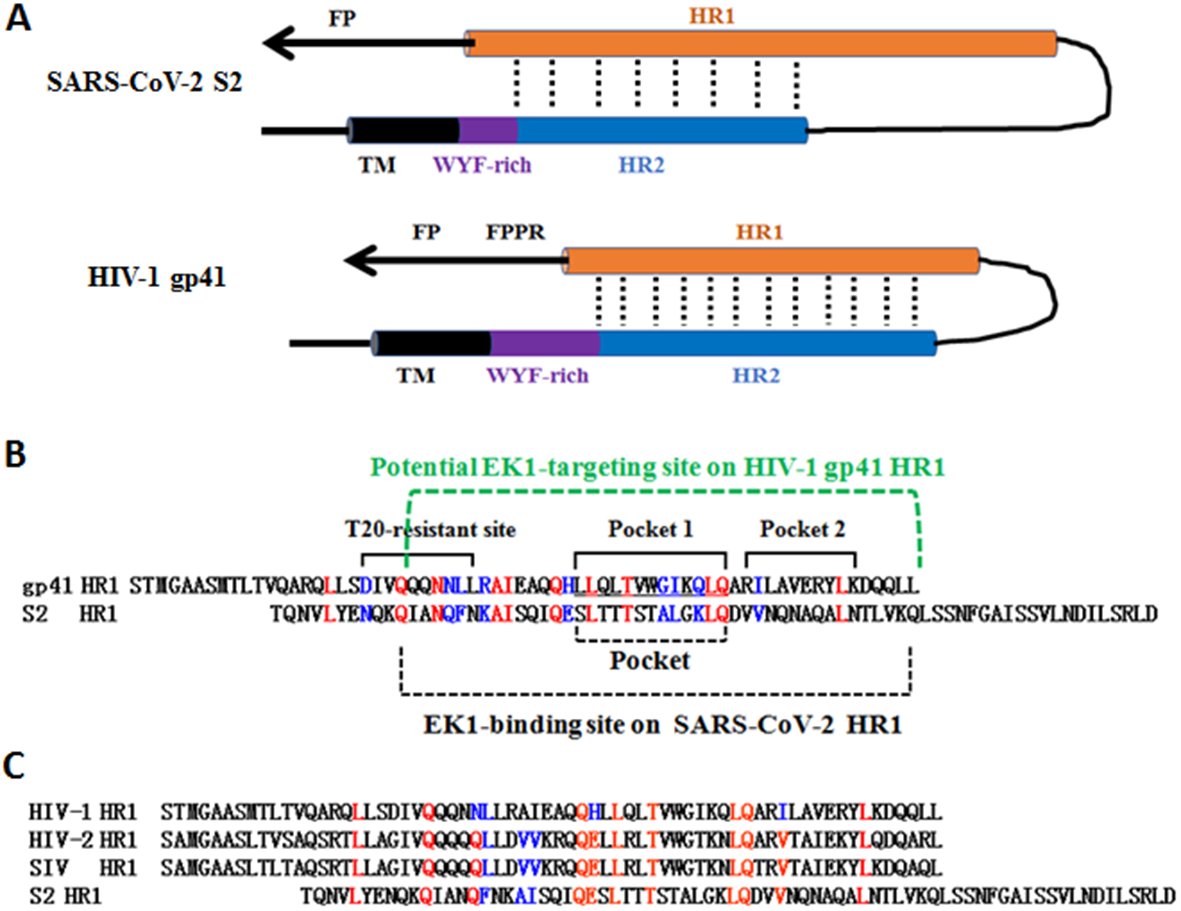

Supplement: Supplemental Material [file TEMI_A_1917309_SM8534.zip › Supplementary files/Figure-S2.tif]

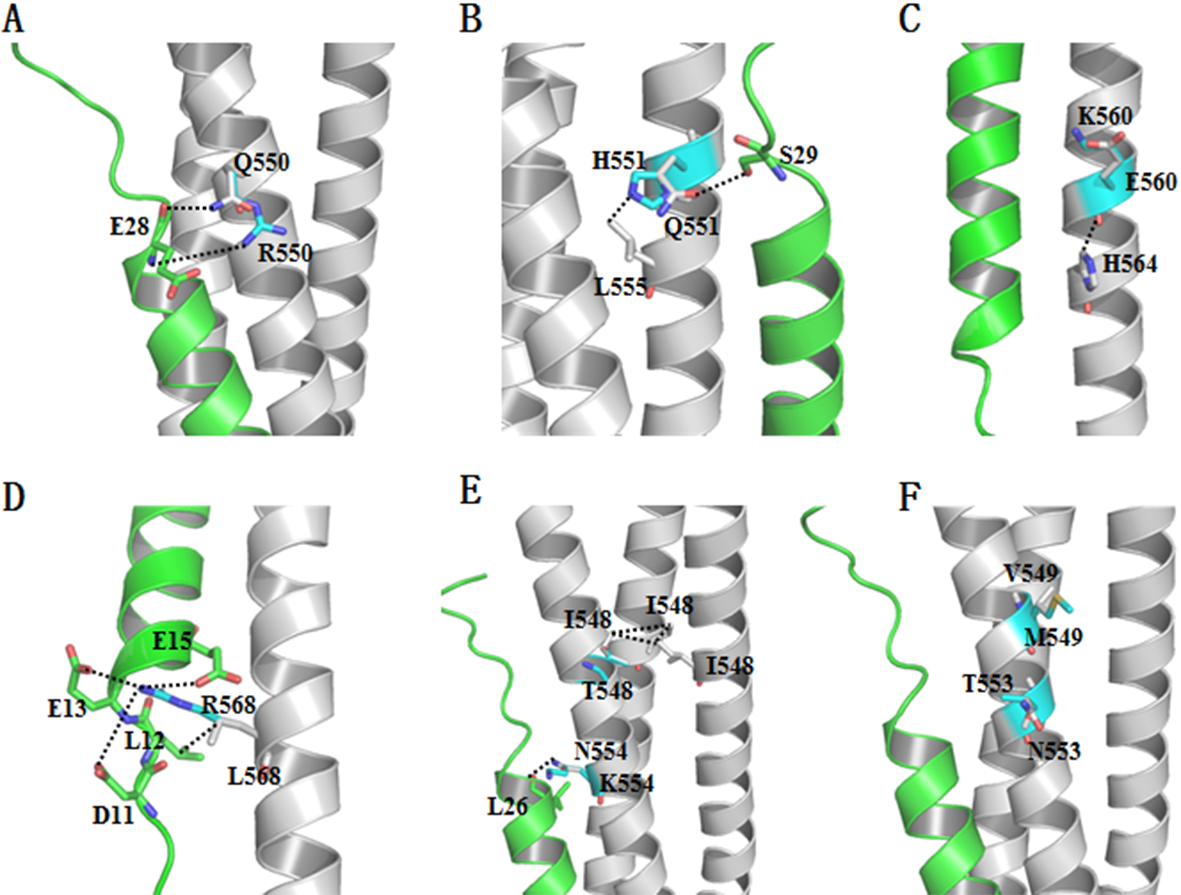

Supplement: Supplemental Material [file TEMI_A_1917309_SM8534.zip › Supplementary files/Figure-S3.tif]
